# A review of surrogate motherhood regulation in south American countries: pointing to a need for an international legal framework

**DOI:** 10.1186/s12884-019-2182-1

**Published:** 2019-01-28

**Authors:** Gloria Torres, Anne Shapiro, Tim K. Mackey

**Affiliations:** 1 Joint Masters Program in Health Policy and Law, University of California, San Diego School of Medicine, San Diego, CA USA; 2 Department of Anesthesiology, University of California, San Diego School of Medicine, San Diego, CA USA; 3 Department of Medicine, Division of Global Public Health, University of California, San Diego School of Medicine, San Diego, CA USA; 4 Global Health Policy Institute, 8950 Villa La Jolla Drive, A124, La Jolla, CA, San Diego, CA 92130 USA

**Keywords:** Surrogacy, Commercial surrogacy, Latin America, Infertility, Advanced reproductive technology

## Abstract

**Background:**

Advances in science and technology coupled with globalization are changing access to and utilization of reproductive health services. This includes the transnational phenomenon of families who use surrogate mothers to reproduce, with forms of altruistic and commercial surrogacy becoming more commonplace. Simultaneously, changes in law, regulation, and policy are necessary to protect surrogates, intended parents, and resulting children. These developments have been slow to adapt to challenges inherent to surrogacy arrangements, most specifically in low-and middle-income countries, including in South American countries.

**Methods:**

We conducted an interdisciplinary non-systematic literature review and legal analysis of existing and pending policy, laws, and regulations related to commercial surrogacy arrangements in Argentina, Bolivia, Brazil, Chile, Colombia, Ecuador, Paraguay, Peru, Uruguay, and Venezuela. The review focused on articles that discussed topics of domestic and international law, policy, regulation, and governance related to commercial surrogacy. We queried PubMed, JSTOR, and Google Scholar for Spanish and English-language articles limited to those published between 2000 and 2016.

**Results:**

Our literature and legal review found a wide variance in how different countries address the issue, including two (Brazil and Uruguay) that have issued guidance attempting to clarify the legality of commercial surrogacy, others who have introduced surrogacy-specific legislation, and a final group with no specific legal mechanisms in place. Our extracted legal case studies also indicate that courts have a hard time interpreting existing law and its applicability to surrogacy. The influence of Catholicism also played a role in the adoption of surrogacy and other advanced reproductive technology (ART)-related legislation.

**Conclusions:**

Changes in global infertility rates, the emergence of new family structures, and advancement of ART are factors that will influence future development of legal frameworks addressing surrogacy in South America. Despite a growing transnational market for commercial surrogacy in many of the countries examined, the current patchwork of policy does little to clarify what forms of surrogacy are legally permissible, nor do they adequately protect surrogates, intended parents, or the children themselves. This points to an urgent need for regional legal and policy harmonization specifically designed to align with public health and human rights principles.

**Electronic supplementary material:**

The online version of this article (10.1186/s12884-019-2182-1) contains supplementary material, which is available to authorized users.

## Background

In vitro fertilization (IVF) has emerged as one of the most promising solutions to the problem of global infertility. The World Health Organization (WHO) defines infertility as “a disease of the reproductive system defined by the failure to achieve a clinical pregnancy after 12 months or more of regular unprotected sexual intercourse.” During IVF, eggs are harvested from a female and fertilized by sperm outside the womb. After fertilization, a selection of the resulting embryos are transferred to a womb [1]. As originally intended, this technique was developed to help women conceive and carry a baby to term. The world’s first IVF baby, Louise Brown, was born on July 25, 1978, in the United Kingdom [2]. The world’s second and India’s first IVF baby, Kanupriya, (alias Durga) was born 67 days later October 3, 1978. [3] Later, in 1986, a court in the state of New Jersey recognized the legitimacy of the traditional/genetic surrogate mother for the very first time in the well- known “Baby M” case [4].

Surrogacy is an arrangement through which a surrogate mother bears and delivers a child for another couple or person [5]. A traditional/genetic surrogacy is when a surrogate is artificially impregnated with sperm, usually from the intended parent, but may also be donor sperm, with the intention of that sperm fertilizing her (the surrogates) egg, thereby making her both the genetic and gestational mother [5]. In gestational surrogacy, embryos that are not genetically related to the surrogate are implanted into the uterus of the surrogate, who will then carry the gestation to term, the intended parent(s) being the individual(s) with ownership of the aforementioned embryos.

Generally, gestational surrogacy is based on the types of contractual arrangements between parties and includes either a commercial or altruistic transaction depending on whether the surrogate receives a financial reward for her pregnancy or not [6]. When individuals or the entities that represent them (e.g. agencies and clinics) enter into any surrogacy arrangement, the laws that can be applied to that arrangement will be from the country where the contract originated and also the country where the baby is born. This explains why commercial surrogacy contracts can be common in countries without surrogacy regulations.

Gestational surrogacy has grown in popularity in different parts of the world over the last two decades (though there is a lack of evidence of increases in age-specific rates), and this increase can be attributed to rates of infertility and changes in traditional family structures [7]. In 2010, 1.9% of women aged 20 to 44 were unable to achieve their first live birth (primary infertility), and 10.5% of women with a previous live birth were unable to have a second child (secondary infertility) [8]. Concomitantly, single parents or same-sex couples have added relevancy to assisted reproductive technologies (ARTs), such as in vitro fertilization, oocyte and embryo donation, and surrogacy [9].

Adoption and surrogacy are two discrete options for people who cannot carry their own children. Over the past several years, many countries now permit same-sex couples to marry and adopting or using a surrogate can be the next step to having a family (though the growth in children raised by same sex couples may also be impacted by artificial insemination by donor sperm, children born into heterosexual unions prior to same sex family formation, or other informal means.) For example, in the United States, it is projected that between 2 and 3.7 million children have a lesbian, gay, bisexual, or transgender parent; approximately 200,000 children are being raised by same-sex couples [10]. This number reflects both adoption and surrogacy arrangements. However, declines in the U.S. rate of adoption and overall rate of international adoptions along with the continued progress and success rates of types of ARTs, may also impact whether gestational surrogacy is viewed a viable or popular pathway to reduce infertility or for same-sex couples who cannot conceive.

Importantly, a global governance framework to harmonize commercial surrogacy laws, regulations, and policies does not currently exist, which has led to widespread variation in how commercial surrogacy arrangements are regulated. Some countries, such as Georgia, India, Russia, and Ukraine, legally allow both commercial and altruistic surrogacies [4]. In contrast, the United Kingdom, Australia, and Canada consider altruistic surrogacy legal, but have banned forms of commercial surrogacy [4]. France, Italy, Germany, and China prohibit both forms of surrogacy [4]. The United States does not have national commercial surrogacy legislation, but in some states, like California, the practice is legal and regulated [11].

Consequently, several academic commentators and human rights organizations have advocated for greater regulation and oversight, while at the same time, religious organizations and feminists’ groups, among others, support prohibiting gestational surrogacy [12]. As an illustration, the European Parliament’s 2014 Annual Report on Human Rights and Democracy condemned the practice of commercial surrogacy and deemed the utilization of gestational surrogates as reproductive exploitation. The European Parliament also declared that commercial gestational surrogacy should be prohibited in order to protect vulnerable women in low income countries [13]. This includes potential alignment with international instruments such as the Convention on the Elimination of All Forms of Discrimination Against Women and the Convention on the Rights of the Child.

Previous studies have examined surrogacy legal and policy arrangements in detail, examining countries such as the United States, Australia, India, and the Ukraine [14–16]. Many studies have scrutinized commercial surrogacy in India and Western Europe, but only few studies have specifically examined laws, regulations, and policies associated with commercial surrogacy in South American countries, despite the fact that the region has become a reproductive tourism destination during the past decade [7]. To address this gap of knowledge, this article conducts an in-depth review and analysis of the policy environment for commercial surrogacy in South America.

Specifically, our review primarily focused on assessing different policy responses to commercial surrogacy in South American countries, analyzing their current legislation, and depicting how regulation has changed over time. For clarification and for the purposes of this study, South America includes the countries Argentina, Bolivia, Brazil, Chile, Colombia, Ecuador, Paraguay, Peru, Uruguay, and Venezuela.

## Methods

We conducted an interdisciplinary multi-lingual literature review examining journal articles, original research, legal cases, law review articles, commentaries, and news reports indexed in three scholarly databases. We queried key search terms on PubMed (Medline), JSTOR, and Google Scholar for Spanish-language articles that contained the words “surrogacy,” “maternidad surrogada,” “gestacion por sustitucion,” “vientre de alquiler,” and “maternidad intervenida,” and English-language articles that contained the words “surrogacy,” “transnational surrogacy” “commercial surrogacy,” “gestational surrogacy,” “reproductive tourism,” and “surrogate parentage” in the Title/Abstract field in the respective databases’ advanced search settings. The review was limited to articles published between 2000 and 2016.

The review focused on articles that discussed topics of domestic and international law, policy, regulation, and governance related to commercial surrogacy. The subject focus necessitated excluding literature that: (1) discussed surrogacy in non-Latin American jurisdictions; (2) articles primarily or solely discussing other reproductive health and medicine issues (e.g., abortion, IVF, gamete and human embryo donation); (3) articles focusing on the bioethical components or arguments for or against surrogacy that did not include discussion of policy and law; and (4) studies focusing on clinical practice and outcomes associated with surrogacy arrangements. Although these other categories are important, this review primarily focused on assessing different policy responses to commercial surrogacy in South American countries, analyzing their current legislation, and measuring how regulation has changed over time.

To supplement information contained in the peer-reviewed literature, the review also examined information sources from the gray literature, which included technical reports or guidance from government agencies, news reports from media outlets (e.g., nonscientific sources), information from nongovernmental organizations or advocacy organizations, pertinent pending and enacted domestic legislation, legal cases, and information from government agency websites.

Examining both the academic literature and gray literature allowed us to identify each South American country’s laws, regulations, and policies related to surrogacy and also assess surrogacy case studies that have been adjudicated in the courts. This allowed us to identify case studies for certain countries that illustrate real-world examples of surrogacy arrangement and the unique legal challenges they face in courts. Case studies were selected on the basis of adjudication of a surrogacy arrangement, legal precedence, and relevance to national surrogacy law. Although this literature review was comprehensive it was not a systematic review.

## Results

### Infertility in South America

South America is not a homogenous region. Important differences exist between the various countries in terms of size, income per capita, demographics, and natural resources, among others. In contrast, shared regional conditions are economic inequalities and high presence of Catholicism and the influence of the Catholic church significantly impacts reproductive issues [9].

Throughout the past three decades, South American societies and their political and economic structures have weathered significant changes and uncertainties, such as destabilization of political institutions, market inflation, and social, economic, and health inequalities [17]. Women are increasingly better educated and now are part of the workforce; combined with socioeconomic conditions, they have delayed their decisions to enter motherhood, which has contributed to a general decline in fertility rates [17].

A 2012 study published in *PLOS Medicine* incorporating 277 health surveys systematically analyzed the world’s infertility rate and found that in 2010, Latin America and the Caribbean had an estimated 1.5% primary infertility rate. [8] In comparison, the primary infertility rate was 1.8% in high-income countries, 2.6% in North Africa and the Middle East region, 3.0% in South Asia, and 1.9% globally. [8]. Despite the 1.5% in the region, in 2010, the lowest estimated prevalence of primary infertility occurred in middle-income countries in Latin America (e.g., Peru, Bolivia, and Ecuador, a, 0.8–1.0%.) [8]. In 2010, estimated secondary infertility in Latin American was 7.3% compared to 10.2% globally [8]. Even though the secondary infertility rate in Latin America is higher than its primary infertility rate, it is still the lowest in the world.

Despite low infertility rates relative to the rest of the world, ARTs use in the region appears to be growing. The Latin American Network of Assisted Reproduction (REDLARA) reported that in 2010 Argentina had 8336 ART procedures compared an increase to 14,980 in 2014 [18]. The same occurred in Brazil in 2010 which had 17,673 ART procedures compared to 27,269 in 2014. Chile had 1652 in 2010 and 3791 in 2014 (Tables 1 and 2). Also, REDLARA reported that a total of 128,245 children were born in Latin America by means of ARTs, from 1990 to 2012 [18].

**Table 1:** ART procedures reported to RLA in 2010

Country	Number of Clinics	Assisted reproductive techniques				Total
		IVF(*)	ICSI(*)	FET	OD(**)	
Argentina	22	810	4462	1231,	1832	8336
Brazil	56	542	12,913	2515	1703	17,673
Chile	7	143	1088	255	159	1652
Colombia	8	358	420	127	284	1189
Ecuador	5	61	248	79	104	492
Peru	4	436	713	104	805	2058
Uruguay	2	32	226	46	48	352
Venezuela	6	320	330	122	331	1103

include 7 cycles of GIFT/TOMI in Chile and 1 in Argentina (*) initiated cycles; (**) includes the transfer of fresh and frozen embryos

Source: Assisted reproductive technologies in Latin America: The Latin American Registry, 2010. BRA Assist. Reprod. | V. 16 | no6 | Nov-Dec / 2012

**Table 2:** ART procedures reported to RLA in 2014

Country	Number of Clinics	Assisted reproductive techniques				Total
		IVF/ICSI	FET	Fresh OD	OD(FET)	
Argentina	24	9083	2903	1826	663	14,980
Bolivia	3	430	41	86	13	576
Brazil	54	16,474	6877	1728	943	27,269
Chile	9	2111	881	461	188	3791
Colombia	11	1196	289	246	102	1868
Ecuador	6	663	200	228	59	1318
Peru	7	1286	445	742	478	3384
Uruguay	7	317	78	84	22	514
Venezuela	7	1119	13,545	7829	3365	1770

Source**:** Assisted reproductive technologies in Latin America: The Latin American Registry, 2014. JBRA Assist. Reprod. | V. 21 | no3 | July-Aug-Sep / 2017

### Key characteristics of Surrogacy law and policy in South America

South American countries have a common legal system. All countries follow a codified civil law system, which means each of the countries conforms to a comprehensive set of legal rules that can be updated and contain all matters which can be brought before a court in a legal proceeding, such as definition, applicable procedure, and sanction or penalty for each action [19]. In the South American legal system, the judge must establish the facts of each case and apply a set of provisions related to the case [19]. A judge makes the final decision; no jury trials exist. In the specific case of surrogacy contracts, most judges apply Civil Law Code; that is the code that regulates general contracts. Due to the lack of specific regulation for surrogacy contracts, the judge must apply the general theory of contract (which generally stipulates that in the absence of legislation, conflict between the parties must be judged the same as a dispute with any other commercial contract/transaction) law to surrogacy disputes. If the surrogacy is between people from different countries, private international law is applicable. In some countries like Colombia, when the matter encompasses fundamental rights, cases are decided by the Constitutional Court.

In addition to the legal system’s involvement, politics and law in South American countries are still considerably influenced by the Catholic Church, especially in the Civil Law system which is originally based on Roman law. Additionally, many of these countries were once Spanish colonies, except Brazil, which was a Portuguese territory. As a result, most laws are compatible with the Roman Catholic tradition, which expressly prohibits use of any ARTs because an embryo is considered to be a person and therefore cannot be manipulated [9]. This includes surrogacy because the practice is also categorized and banned as an ART [20]. Generally, for surrogacy to be regulated, it must be included in a country’s legislative code, with codes generally enacted by the state’s Congress, though often never being enacted due to the socially controversial nature of ART [9].

Table 3 contains a brief overview of each country’s situation in terms of infertility rate, number of infertility clinics, and the legal status of surrogacy arrangements as detailed in our country-by-country analysis below.

**Table 3:** Infertility data and surrogacy legislation status in South American countries

Country	Infertility rate 2010*	No. Fertility Clinics**	Surrogacy legislation status	Active Legal instrument	Pending Bill
Argentina	1.8%	22–25	Unregulated		
Bolivia	0.9%	Uncertain	Unregulated		PL 185–2001/2002
Brazil	1.6%	56	Guidelines: Altruistic surrogacy is allowed	Resolution No. 2.013/13 Federal Medical Council	
Chile	1.8%	7–9	Unregulated		No. 6306/07
Colombia	1.5%	8	Unregulated		
Ecuador	1.0%	5	Unregulated		No. 261836/16
Paraguay	1.9%	1–3	Unregulated		
Peru	0.8%	4	Unregulated		No. 2839/2013-CR
Uruguay	2.0%	2	Regulated: Altruistic surrogacy is allowed	Ley 19.167/2013	
Venezuela	1.2%	6	Unregulated		

*prevalence of primary infertility among women aged 20–44 years

Source: Assisted reproductive technologies in Latin America: The Latin American Registry, 2010. BRA Assist. Reprod. | V. 16 | no6 | Nov-Dec / 2012

### Country-level Surrogacy Laws, policies, and case studies

#### Argentina

Argentina has an estimated 60,000 couples with infertility, this number includes male infertility [21]. While 1.8% of the female population is diagnosed with infertility, only a small percentage sought fertility treatment in 2010 [21]. With the introduction of public health care coverage for infertility the number of ART procedures has been increased significantly (Tables 1 and 2). Argentina also has the most well-established private fertility industry in South America, offering IVF and surrogacy services not only for residents but also for foreigners traveling to seek infertility treatment. During the past decade, Argentina has become an affordable destination for fertility tourism [22].

In 2010, Argentina regulated ART for the first-time, recognizing a person’s right to procreate as a fundamental right and categorizing infertility as an illness. The law, “Ley 14.208/2011” was sanctioned in 2010 but was applicable only in the Buenos Aires province [23]. In 2013, with Ley 26.862/13, ART access was extended to any adult person regardless of his or her age, marital status, and infertility situation, allowing national access to ART [24]. Fertility services were covered mostly through out-of-pocket payments until these legal provisions were enacted. Currently, public or private health insurance plans, donations, and the government finance fertility treatments [25].

Like any other civil law country, Argentina’s civil code covers all contracts and family law regulations. Despite this, Catholic groups and some lawyers consider surrogacy contracts void because they are interpreted to deem a contractual subject matter that is immoral and against good customs (Argentinian Civil Code Art. 386). Additionally, it is argued that carrying a baby and giving it away cannot be subject to contract because children should not be the objects of an economic transaction. Because contextual ambiguity persisted and surrogacy contracts were not technically illegal, the practice occurred with legal uncertainty (see Case Study #1 in Additional file 1) and in unregulated spaces of the economy. For example, Internet reports stated Argentinian women were offering their services as surrogate mothers [26].

In response, in February 2011, then Argentinian president Cristina Fernandez signed executive order 191/2011 and created a commission to update and unify the civil and commerce codes. The proposed bill contained several articles concerning surrogacy. “Gestacion for sustitucion” was to be permitted in its altruistic form, and the bill included protections for the surrogate mother [27]. However, the surrogacy articles/provisions were eliminated before the bill was finally approved.

Relatedly, the Civil Code was updated in 2014 (Ley 26.994) and contained a special chapter dedicated to regulating parentage when a child is born through an assisted reproductive technique. The prospective parents were required to sign a consent document before the IVF procedure begins and when the child is born, the gestational mother is considered the legal mother according to law. Subsequently the gestational mother can waive her maternity rights and give all rights to the intended parents.

Later, in November 2016, a Senator for the “Union Civica Radical movement”, Laura Montero, proposed bill 2574/15 that seeks to regulate surrogacy and titled the bill “gestation por sustitucion.” The bill allows altruistic surrogacy but required a judge’s permission before starting the procedure. Conditions include that the surrogate woman should be in good health, already have at least one child, and can be a surrogate woman only twice. Also, the intended parents must reside in Argentina for at least three years prior to the procedure. Finally, the bill includes a three- to six-year prison sentence for any person who acts as intermediary or a medical doctor and carries out the procedure without judicial authorization [28]. This bill was rejected in April 2017.

As a consequence, despite the fact that Argentina may represent an attractive destination for fertility tourism and commercial surrogacy, no regulations exist. One of the goals of the aforementioned bill was to end reproductive tourism in Argentina by adding the three-year residency requirement.

#### B. Bolivia

Bolivia has one of the highest fertility rates in the region: on average, a Bolivian woman has three children during her lifetime, [29] compared with Chilean or Brazilian women’s lifetime average of 1.8 children [8]. Nevertheless, according to the most recently available national demographic census, Bolivia’s infertility rate was 10.7% in 2008 [30]. Infertility treatments are not covered by the national health care system, but instead must be paid out-of-pocket [31].

Bolivia also does not have any legislation that pertains directly to commercial surrogacy, better known as “maternidad delegada.” Lack of regulation does not mean that commercial surrogacy arrangements do not take place in the country, even if surrogacy statistics are not available. In fact, it has been reported that Bolivia has become an active player in the commercial surrogacy industry. For instance, a 2014 study found that in 70% of the surrogacy cases, the intended parents are foreign [32]. In the absence of regulation, fertilization procedures hinge on the ethics of doctors performing such procedures at fertilization clinics. Poverty is cited among the chief reasons that women in Bolivia become surrogate women [32].

In 2001, Bill PL 185–2001/2002 was proposed at the Bolivian National Congress to address infertility and surrogacy issues. It required written consent of all parties involved before starting any fertilization procedure. It stated surrogacy should be viewed as a last recourse and required that the intended parents be in a stable relationship and the surrogate woman be between 18 and 40 years old. The law is not clear whether commercial surrogacy is permitted or not. However, this law prohibits embryos’ commercialization, though there has been no further discussion or passage of the bill since it was originally introduced in 2001 [33].

Hence, Bolivia’s relatively high rates of fertility combined with its classification as a lower middle-income country where women may experience poverty and lack of economic mobility, means that it continues to act as an international destination for transnational commercial surrogacy.

#### C. Brazil

Official infertility rates in Brazil were not readily available when conducting this review, but some studies have discussed a general trend of declining fertility rates over the last three decades [29]. In the latest United Nations fertility report (2015), Brazil had the lowest fertility rate in the region, along with Chile [29]. According to the report, between 2010 and 2015, women from Brazil had an average of 1.8 children [29].

Since 1988, Brazil has operated a Federal Constitution that covers individual and collective rights. Health care is considered a universal right and the government’s duty is to protect this right. Brazil’s Unified Health System (SUS) provides free healthcare access for the entire population but does not provide coverage for all forms of health services. For instance, fertility treatments are currently not covered by SUS [31].

Commercial surrogacy is forbidden based on article 199(4) of the Brazilian Federal Constitution that interprets it as a form of human organ trafficking. Commercial surrogacy is considered uterus trafficking, and consequentially is constitutionally forbidden. The Brazilian Congress has not enacted any clarifying regulation about surrogate motherhood, also known as “Barriga Solidária” or “uterus temporal donation.” Responding to the lack of legislation, the Federal Medical Council created a guideline for altruistic surrogacy that has been in place since 2010 [27], which comprises the only set of rules applicable in Brazil to differentiate this practice from commercial surrogacy [34].

The latest version of this guideline, RCFM No. 2.121/2015, [35] contains the regulations that generally prohibit commercial surrogacy. Altruistic surrogacy is permitted only in two cases: (1) when the intended mother can contribute an egg however, she herself cannot carry a gestation to term or has a medical condition that puts her life at risk; or (2) when the intended parents are part of a same-sex marriage. The surrogate mother must belong to the same family as one of the partners in a consanguineous kinship to the fourth degree (i.e first degree = mother, second degree = sister, third degree = aunt, fourth degree = cousin).

Additionally, temporary uterus donation cannot be profitable. The parties must sign a contract that establishes the child’s parentage and the intended parents must warrant they will pay medical and related pregnancy expenses; additionally, they will be the parents listed on the birth certificate [35]. This resolution is a temporary solution, however, and Brazil is waiting for a legal framework to be enacted by Congress that permanently regulates surrogate motherhood (Case Study #2). Overall, Brazil’s relatively strict legal framework on commercial forms of surrogacy may limit the practice inside and from outside of the country.

#### D. Chile

According to a 2015 report published by the Ministry of Health in Chile, infertility affects 10.4% of women [36]. The Chilean health system is one of the most egalitarian systems in the region [31]. It covers low-complexity fertility treatments such as diagnosis, ovarian stimulation, spermogram, sperm separation, and intrauterine insemination, as well as high complexity treatments such as IVF [37].

Despite access to forms of reproductive health services, Chilean society is very traditional, and values like dignity, faithfulness, and the concept of family are difficult to negotiate, much less legislate [38].

Although church and state are separated, the Catholic Church still has a significant influence on Chilean society, an influence that also extends to public policy and national legislation [39]. Bills that contravene societal and Catholic values are enacted only rarely. For instance, in December 2008, Bill 6306–07 was presented, and contained a single article (item 23) that penalized surrogacy and would punish with imprisonment any intervening party in a surrogacy contract, as well as the intended parents, the doctor, and even the surrogate mother, who also would be required to attend responsible motherhood psychological therapy [40]. This bill is still pending and has not been enacted. In the meantime, surrogacy remains unregulated and judges must apply the law according to a biological test that defines the mother as the person who delivers a child (article 183, Chilean Civil Code).

#### E. Colombia

Catholicism was the official religion in Colombia until 1991, when the Political Constitution was modified and conferred freedom of religion on all citizens. Despite this change, Colombia persists as a country in which the Catholic faith is still the religion of the majority. In deference to this reality, the Colombian Congress avoids regulating issues that are controversial, including abortion and surrogate motherhood. In Colombia, no law exists that makes a surrogacy contract legal or illegal. Yet, the fact remains that infertility affects 6.9–9.3% of all Colombian couples [41].

The Colombian health system is a public service that guarantees the population access to services and financial protection through social insurance [31]. Coverage is universal but does not encompass all services. Fertilization treatments are not yet included in the national coverage, although in 2014, the Constitutional Court (in decision T-528) ordered the Colombian government to add IVF treatment to the Mandatory Health Plan [42].

In stark contrast to mostly regional policy inaction on surrogacy, Colombia was a pioneer in ART; the first in vitro baby in Latin America was born in Colombia in 1985 [43]. Compliant with Article 42–6 of the Colombian Constitution, children born naturally or through ARTs have equal rights and obligations. Concordant with this article, when a woman gives birth to a baby, she is the child’s mother and her name will appear on the birth certificate. If she is married, the law presumes the baby’s father is her spouse, regardless of how she became pregnant.

To resolve the conflict created between parties involved in a commercial surrogacy arrangement, the case must be presented in a court. Additionally, if the intended parents wish to be the legal parents of the child their surrogacy cases must be brought before a judge, the natural mother must relinquish her motherhood and give her rights to the intended parents. However, there is no legal obligation or guarantee that the surrogate mother will forfeit her rights after the child is born.

In recent years, an increasing number of women are offering their services as surrogate women, necessitated by their economic situations, being unemployed, and a dearth of opportunities for social advancement (Case Study #3) [44]. In some cases, being a surrogate woman is an option to accumulate a decent sum of money to buy a house or start a business. The internet and newspapers are common places to advertise surrogacy services. Hence, Colombia’s economic conditions and progressive approach to ART could be factors driving potential transnational surrogacy arrangements.

In 2009, the Colombian Constitutional Court urged Congress to enact a legal framework for surrogacy motherhood, to protect newborns’ rights, women’s rights, and avoid conflicts during a breach of contract between the parties [45]. This decision (T-968/2009) is the only legal precedent for surrogacy motherhood in Colombia (**Case Study #3**). However, Congress has not yet enacted any legislation to clarify commercial surrogacy issues. The ramifications are even more relevant, because in 2014, the same court decided that same-sex couples can adopt a child when just one of them is the biological parent of the child. This Constitutional decision enables same-sex couples to be parents through surrogacy. However, in November 2016, Congresswoman Maria del Rosario Guerra, a member of the right-wing party Centro Democratico, introduced Bill No. 026 of 2016 to the Colombian Congress, which allows altruistic surrogacy and forbids commercial surrogacy; she argued this practice exploits women and violates a minor’s rights. This bill was approved in the House of Representatives but did not pass the first debate at the Senate.

#### F. Ecuador

In Ecuador, infertility affects around 15% of the population [46]. Public health services are universal and the state provides them free of charge (including services such as diagnosis, treatment, medication, and rehabilitation [31].) Assisted reproductive technologies are not regulated and thus not included as coverage in the Ecuadorian health care system. Hence, fertility treatments must be paid out of pocket [31]. However, IVF has been used since 1992 through 11 known fertility centers [47]. No surrogacy cases were presented before a court until 2016.

In September 2016, Congresswoman Maria Alejandra Vicuna, member of the Alianza Pais party, presented a bill to regulate ARTs in Ecuador. Article 6 of this bill allows surrogacy and does not distinguish between altruistic or commercial surrogacy. The sole requirement is that the surrogacy contract should be written to contain the intended parents’ explicit procreation desire and their commitment to assume responsibility for the child-to-be. The legal document must also be signed before a public notary [48]. Vicuna’s bill is still in study but constitutes the most permissive legal stance in the region because most of the countries examined explicitly forbid commercial surrogacy in one way or another.

#### G. Paraguay

Paraguay does not publish official infertility statistics but has one of the highest fertility rates in the region. On average, a woman has 2.6 children during her lifetime [29]. The Paraguayan Constitution has recognized citizens’ fundamental rights to health care and adopted a universal free health care policy in 2008 [31]. Despite this, infertility treatments are not covered by the health care system.

IVF is not in widespread use in Paraguay according to REDLARA. Between 1990 and 2012, Paraguay reported only 12 IVF cases, which represent 0.01% of the procedures in the entire region. A high fertility rate plus limited use of ARTs may be the primary reasons they have no current ART or surrogacy legislation. In the absence of legislation, each fertility center has created its own guidelines applicable to procedures such as IVF or embryo cryopreservation, and doctors determine the quality standards for each procedure [49]. Similarly, surrogacy arrangements are not legislated nor have they been addressed by the courts.

#### *H. Peru*

In Peru, as in other South American countries, it is difficult to accurately report the infertility rate because no official statistics are available, but the rate is estimated at 15% of the fertility-aged population who have been unable to conceive a child after a 12-month period [50]. The Peruvian public health care system covers only low-complexity fertility treatments, made available exclusively to married couples or heterosexual domestic partners. High-complexity fertility treatments, such as IVF, are not covered [51].

No specific surrogacy legislation exists in Peru, except the general law of health (Ley 26,842), which partially addresses the issue. In article 7, the law states that every person has the right to access fertility treatment, or to use ART, but the genetic mother and the gestational mother should be the same person [44]. Hence, the regulation does not allow for an egg’s or uterus’ donation. This legislation is problematic because it is not clear (Case Study #4). Its intent was to recognize the population’s right to procreate and gain access to ARTs, and simultaneously accept traditional surrogacy because the genetic and the gestational mother are the same person.

Even if Peruvian health law does not explicitly permit gestational surrogacy, to make the law enforceable requires sanctions if people’s conduct violates the general principles of the law. The Peruvian Penal Code does not contain any sanctions for infertility treatments, thus the practice is not allowed, but is not explicitly prohibited. This logic follows by applying the reservation principle (article 2, (24) Peruvian Constitution) that states, “No one is bound to what the law does not command or deprive of what it does not prohibit” [51].

This legal loophole allows commission of crimes such as forgery, fraud, and child trafficking [52]. For example, surrogate mothers have delivered babies in hospitals but used the intended parents’ identity, [52] and put false information on the birth certificate, which is a crime. Legislation notwithstanding, Peru has a prominent commercial surrogacy industry. For instance, in 2006, reports cited a surrogacy network working from Spain using Peruvian surrogate mothers, and it took two years for the police to finish its investigation and break up the network [53].

Additionally, it is easy to find online ads offering surrogacy services in Peru [54]. To bridge this regulatory gap, on October 30, 2013, Bill No. 2839/2013-CR was introduced to regulate surrogate motherhood and only allow altruistic surrogacy. This initiative seeks to modify Article 7 of the General Law of Health, [55] and has been in discussion for years without either being enacted or rejected. Despite these attempts at legislation, Peru remains an international destination for commercial surrogacy, given ambiguity and gaps in current laws that have yet to be closed.

#### I. Uruguay

Uruguay is a small country with a population of 3,467,054 people as reported in its 2015 census [56]. The infertility rate among this population is estimated to be between 15 and 18% [57]. One of the most liberal countries in the region, Uruguay’s Parliament approved legislation addressing ARTs (Ley 19.167/2013) in 2013, [58] which included ART coverage in the Uruguayan public health care system (Article 3). Article 25 makes special reference to gestational surrogacy, stating that surrogacy contracts are void except in cases when the intended mother has an illness that impedes her ability to carry the gestation to term. In these cases, the intended parents have their embryos implanted into a surrogate mother who should be a second-degree relative of either member of the couple, and the surrogacy arrangement should be altruistic [58]. The statute is innovative because it solves the issue normally created with surrogacy contracts in Latin America by allowing the intended parents’ names to be on the birth certificate without needing to appear before a court.

#### J. Venezuela

Venezuela’s unofficial infertility rate is between 10 and 15% [59]. The Venezuelan National Public Health System (SPNS) does not cover fertility treatments; however, gratis care and universal availability are fundamental principles of the public health care system [31]. Neither surrogacy nor ARTs have any specific applicable legislation. Judges’ decisions interpret existing laws contained in the civil code, the Venezuelan Constitution and Supreme Tribunal rulings, which offer a possible legal framework to settle surrogacy conflicts [60].

For instance, Venezuela’s Civil Code states an adjudication of maternity is determined by who gave birth to the child, regardless of the genetics [60]. On the other hand, article 56 of the Constitution recognizes the fundamental right to know one’s biological or genetic origin (Case Study #5) [61]. Furthermore, decision No. 1456 of the Supreme Tribunal of Justice mentions the concept of procreation will (the explicit desire to procreate) [61].

Hence, judges must interpret existing laws by applying the delivery test, genetic test, and the procreation-intent tests from disparate sources of legal jurisprudence. Venezuela has neither a regulation or bill in process, leaving all surrogacy decisions dependent upon the judge’s values and his or her interpretation of the law. Although interpretation can generate a decision, such decisions are not uniform because they depend on how much weight the judge prefers to give each piece of legislation.

## Discussion

The objective of this article was to review, characterize and describe the legal status of surrogacy arrangements in South America. Results indicate that South American societies and public views regarding ART and surrogacy have changed over time, but regulations and laws have been slow to adapt, which creates complicated situations illustrated by the varying laws and case studies. South America, like other regions of the world, regulates or fails to regulate surrogacy arrangements and legislates it in different and disparate ways, often in relation to whether the situation relates to a commercial or altruistic surrogacy arrangement. For example, commercial surrogacy arrangements are expressly prohibited in some countries but also remain unregulated in many countries. Further, we did not observe any laws in place that regulate the maximum number of embryos that may be carried by a surrogate woman, nor the gestational age of the surrogate woman, nor the need for counseling.

In one group of countries, including Argentina and Colombia, legislative bills were introduced and then rejected. In another group (Bolivia, Chile, Ecuador, and Peru) legislative bills have been introduced to specifically regulate surrogate motherhood. However, none of these bills have reached a final conclusion, and either remain pending, under discussion, or in legal hiatus prior to a final decision to enact or deny such legislation. Conversely, Brazil - which has generated guidelines - and Uruguay are the only South American countries that expressly allow altruistic surrogacy. In the remaining countries reviewed, Paraguay and Venezuela have not specifically addressed surrogacy in any way, but have existing legislation that can be interpreted to help judges make decisions about these arrangements. These results demonstrate a great deal of regional variability in how national legal frameworks, guidance instruments, and regulations address surrogacy in South America.

Surrogacy has not been regulated in Latin American countries because the traditional Catholic belief system plays a preeminent role in the judicial system and what public policy is legislated. This despite most South American countries’ constitutions upholding the separation of church and state. Introducing and passing legislation about controversial topics, such as ART and surrogate motherhood, generates a huge amount of public debate and controversy, because a large segment of the population believes human procreation should be limited to the natural ways dictated by the *Donum Vitae* (gift of life), a 1987 document written by Joseph Cardinal Ratzinger (former Pope Benedict XVI). It details the Catholic Church’s position on the dignity of human life, and addresses specific biomedical ethical issues about respect for human life, technical interventions into human procreation, and the status of human embryos and fetuses [20]. This document expressly bans ARTs [20].

Beyond the broader challenges of global infertility, new family structures make the issue even more complex, particularly same-sex marriage that is now legal in 24 countries worldwide, including four in South America (Argentina, Brazil, Colombia, and Uruguay). Other countries in the region have approved domestic partnerships between same-sex couples (Chile and Ecuador) [62]. Accordingly, surrogacy and adoption options are offered to couples experiencing infertility and same-sex couples who would like to start a family, a trend that will likely impact future legal developments on regulation of surrogacy in the region.

The most pressing concern is the growing and ever-expanding transnational market for commercial gestational surrogacy that uses surrogate mothers from low-income countries where disparities due to gender, class, race, and ethnic hierarchies are prominent and have become an economic motivating factor in the growth of reproductive tourism [63]. In the case of domestic surrogacy, the intended parents’ income is often three times that of the surrogate women creating potential ethical issues and power imbalances in the ability to negotiate a surrogacy contract or dispute in relation to the surrogacy arrangement [63].

Fertility clinics are also taking advantage of current legal loopholes and/or lack of regulation. Yet, lack of regulation or regional regulatory coherence does not appear to be an obstacle if women wish to offer their services as a surrogate mother, because the Internet and social media have emerged as popular methods to market and solicit surrogacy services, even when the practice is not legally protected or permitted. The Internet has been reported as a common forum for prospective surrogates and clients (regardless of location or citizenship) to offer or request surrogacy services [64]. South America’s maturing reproductive tourism market is fortified by the lack of consistent regulation and pervasive poverty indicators. The problem exists between the market and in-country legislators seeking to follow moral and appropriate customs dictated by tradition and religion.

Multiple authors cited in this article [26, 27, 65] expressed their concerns about the lack of effective legislation, and agreed that more robust legal frameworks are necessary to regulate commercial surrogacy. For instance, in February 2016, a group of experts on parentage and surrogacy met at The Hague Conference on Private International Law. The meeting was attended by 21 experts representing 21 states globally, including some states involved in international surrogacy arrangements. Conference participants reached the conclusion that an international public policy framework for surrogacy agreements, which focuses on protecting children’s legal status and that prevents exploitation of women surrogates, is urgently needed [66].

Creating an international public policy framework is likely an ambitious goal, but the World Health Organization—more specifically the Pan American Health Organization (PAHO) that exerts influence over public health issues in South America—could take a leadership role in creating consensus and guidelines to harmonize commercial surrogacy policies. These policies should be aligned with public health principles while also acknowledging the unique values and culture of the region when establishing rules to protect all intervenient parties in a surrogacy agreement. Many decisions are pending, and answers to such questions such as whether there should be requirements for the age of surrogate women, how many times an individual can act as a surrogate woman, how many eggs should be fertilized, what sort of economic compensation is ethical, and which state is obligated to offer citizenship to the newborn if the surrogacy crosses borders, are questions that will have to be addressed in such a framework.

## Conclusion

Reproductive tourism in South America is a reality to be reckoned with; an increasing number of fertility centers and clinics selling surrogacy services offer surrogate mothers from South America to locals and foreigners, revealing the urgent need for surrogacy regulation and pointing to an opportunity for a regional legal or governance framework that could be developed with the leadership of PAHO, a respectable international organization in the region. The framework could serve to accelerate local policymakers’ decision-making about commercial surrogacy, aid in applying consistent legal principles in the judicial process of surrogacy court cases, and should be designed to protect all parties, with a special emphasis on vulnerable groups, such as the children and surrogate mothers.

## Additional file

Additional file 1: Case Studies.Text boxes of country surrogacy legal case studies. (DOCX 26 kb)

## Abbreviations

ART: Assisted Reproductive Technologies
FET: Frozen Embryo transfer
ICSI: Intracytoplasmic sperm injection
IVF: In vitro fertilization
OD: Oocyte donation
PAHO: Pan American Health Organization
PL: Public Law
REDLARA: The Latin American Network of Assisted Reproduction
