# Supplementary material for: A review of surrogate motherhood regulation in south American countries: pointing to a need for an international legal framework
Source: BMC Pregnancy Childbirth. 2019 Jan 28;19:46. doi: 10.1186/s12884-019-2182-1 (PMC6350392; doi:10.1186/s12884-019-2182-1)
Supplement: Supplementary file 1 — Case Studies.Text boxes of country surrogacy legal case studies. (DOCX 26 kb) [file 12884_2019_2182_MOESM1_ESM.docx]

**Additional file 1 – Case Studies**

**CASE STUDY #1—**Before the Civil Code was modified in 2014. A male same-sex married couple hired an Indian surrogate mother. When the baby was born, the Argentinian couple tried to get the child’s birth certificate through the Argentinian consulate in India, but their petition was denied because Argentinian law required that the birth certificate contains the mother’s name. Indian law estab­lishes that when a child is born of a surrogate mother her name will not appear on the birth certificate. In a 2012 appeal, an Argentinian judge granted the couple the right to register the baby with the two fathers’ names. This case set an important precedent in Argentina because it was the first step in recognizing that legal parenthood does not need to include the surrogate woman, but rather the intended parents when assisted reproductive techniques were used [27].

**CASE STUDY #2—** On January 29, 2012, the first child registered to two fathers was born in Brazil. The surrogate woman was a cousin of one of the two fathers and was an altruistic surrogate woman to help the same-sex couple become parents, representing an important precedent in the country (she was a gestational surrogate) [28]. Another more recent case occurred in September 2016, a 44-year-old woman, gave birth to her first grandson. She decided to be a gestational surrogate mother for her son who the year before had married another man, who is the baby’s biological father and the mother was an anonymous donor. The couple lives in France where surrogacy is prohibited by law [37].

**CASE STUDY #3—** In 2006, a married couple who were U.S. residents decided to travel to Colombia for in vitro fertilization. After three unsuccessful attempts, they decided to hire a healthy young woman to carry their embryo. The surrogate mother was a hard-working woman in a precarious economic situation and the pregnancy was not viable. Still determined, the couple decided to try traditional surrogacy instead, when the surrogate is also the biological mother. At last, the Colom­bian surrogate got pregnant with twins, and she and the American couple made an oral contract in which the surrogate agreed to release the babies to their biological father after delivery. But as time passed, she changed her mind and decided she wanted to keep the twins. The father initiated a legal battle, arguing that the twins would have better opportunities with him and his wife living in the United States rather than living in a low-income situation with their biological mother. A judge decided to give the father temporary custody of the twins when they were nine months old, and subsequently they traveled to the United States and separated from their biological mother. The Colombian Constitutional Court, in decision T-968/2009, applied the law stating children’s’ rights had priority over other people's rights and decided that the biological mother must be part of the twins' lives because a child should not be separated from a mother especially during early infancy. The Court expressed that poverty was not a valid reason to separate them from their mother and recognized the surrogate mother as the biological mother. The twins remain in the United States with their biological father and his wife, but the father must travel with the children to Colombia three times a year. The biological mother is permitted to visit the twins in case of illness and to enable permanent contact with them.

**CASE STUDY #5—** In 2012, a Venezuelan girl was born through (gestational) surrogacy, and in compliance with the civil code, the surrogate mother was the legal mother and her name was listed on the birth certificate. The intended (genetic) parents went before the court to claim that the birth certificate should be modi­fied to substitute their names. On January 9, 2013, the Judge ordered the baby girl’s birth certificate to be changed by applying Constitution article 56 that recognizes the right to know one’s genetic origin and recognized the rights of the biological or genetic parents [63].

**CASE STUDY #4—** The Peruvian Supreme Court settled the first surrogacy case by deciding in favor of the intended parents during a case involving family members. A couple wanted to have children, but the intended mother was unable to get pregnant. They decided to ask another couple (the husband’s brother and sister-in-law) for help. Through IVF using the intended father’s sperm, and the sister-in-law’s egg, the sister in law became pregnant creating a traditional/genetic pregnancy. During the pregnancy, the sister-in-law and her husband received periodic payments. Once the child was born, the intended parents took care of the baby and started the adoption process. At some point before the adoption was finished, the biological mother (sister-in-law) changed her mind, and brought the case to a judge to recover the custody of the child. The Court ruled that the child should be in the custody of the adoptive parents because they had been most interested in caring for the baby since the beginning of the arrangement. The biological mother appealed to the highest court, but the judges determined (in Cassation No. 563-2011-Lima) that a child conceived by assisted fertilization had the right to have an ideal family situation. According to the Court, the child’s rights prevailed over the rights of the biological mother and her husband to exercise their parental rights, especially when both had agreed to procreate in exchange for economic benefits. The intended parents retained the child's custody. This decision was an important precedent in Peru because the court recognized not merely the reality that surrogacy is occurring in their jurisdiction, but that an explicit procreation desire trumped the biological test that in most jurisdictions is determinant to decide who has right over a child.
